# Gender differences in sore throat and hoarseness following endotracheal tube or laryngeal mask airway: a prospective study

**DOI:** 10.1186/1471-2253-14-56

**Published:** 2014-07-19

**Authors:** Maria Jaensson, Anil Gupta, Ulrica Nilsson

**Affiliations:** 1 Division of Anaesthesiology and Intensive Care, Örebro University Hospital, Örebro, Sweden; 2 Schools of Health and Medical Sciences, Örebro University, Örebro, Sweden

**Keywords:** Sore throat, Hoarseness, Endotracheal tube, Laryngeal mask airway, Postoperative complications

## Abstract

**Background:**

Postoperative sore throat and hoarseness are common minor complications following airway manipulation. This study was primarily done to determine gender differences in the incidence of these symptoms and the location of POST after laryngeal mask airway (LMA) and endotracheal tube (ETT).

**Methods:**

A total of 112 men and 185 women were included during a four month period. All patients were evaluated postoperatively and after 24 hours about the occurrence of sore throat, its location and hoarseness. If the patients had any symptom, they were followed-up at 48, 72 and 96 hours until the symptoms resolved.

**Results:**

There was no significant gender difference in postoperative sore throat (POST) and postoperative hoarseness (PH) when analyzing both airway devices together. The incidence of sore throat and hoarseness were higher postoperatively after an ETT than an LMA (32% vs. 19%, p = 0.012) and 57% vs. 33% (p < 0.001) respectively. Significantly more women than men had POST after an LMA (26% vs. 6%, p = 0.004). No significant gender difference was found in either POST or PH after an ETT or in the incidence of PH after an LMA. More patients located their pain below the larynx after an ETT vs. an LMA (24% vs. 4%). Pain above the larynx was more common after an LMA than an ETT (52% vs. 37%).

**Conclusions:**

In a clinical setting where women are intubated with a smaller size ETT than men, there were no significant differences in POST or PH between genders. Additionally, more women than men have POST when an LMA is used. Awareness of POST and PH may help streamline patients in whom the best airway device could be used during anesthesia and surgery.

## Background

Postoperative sore throat (POST) and hoarseness (PH) after general anaesthesia are common but minor adverse events after endotracheal intubation (ETT) and laryngeal mask airway (LMA). A systematic review found a lower incidence of airway morbidity following the use of an LMA compared to an ETT [1].

Previous studies have shown that women are at a greater risk for POST compared to men after an ETT [2-4]. There is no consensus in the literature today as to what constitutes POST, and how or when it should be measured [2,5-7]. Therefore, the results between studies may not be comparable [8]. Another contributing factor towards the variation in results could be that different ETT sizes have been used in women: size number 6.5 [4], number 7.0 [9] or 7.5 [2] while some authors do not state what ETT size was used [3]. Since patient satisfaction with anaesthesia may be further improved by reducing the risk of POST and PH [10,11] the symptoms needs to be continuously re-evaluated in different settings. The appropriate ETT size in women and men is still unclear and debated in the anesthesia community [12]. However, there seems to be some evidence that women benefit from a smaller size ETT [13]. The etiology of POST is also not clearly understood, but it appears to be an inflammatory process since the tracheal mucosa has been found to release inflammatory mediators after intubation [14]. However, the exact anatomical location of sore throat still remains uncertain in patients [8]. Although the evidence points towards female gender as a risk factor for POST, there have been few studies evaluating the difference between genders in recent years [15].Therefore we sought to determine if there are any gender differences in relation to the incidence of sore throat and hoarseness following endotracheal intubation and laryngeal mask airway.

This study was done with the primary aim to determine if there is a gender difference in the incidence and location of postoperative sore throat, and for the incidence of hoarseness after a laryngeal mask airway and endotracheal tube. We also determined the duration or persistence of these symptoms.

## Method

This investigation was a non-randomized, prospective and longitudinal study performed at the Örebro University Hospital, Sweden. After approval from Regional Ethics committee in Uppsala, Sweden (nr. 2012/392), informed written and verbal consent were obtained from all patients. The patients were either inpatients or ambulatory patients.

The inclusion criteria were: age >18 years, elective surgery with either a laryngeal mask airway or endotracheal tube. The exclusion criteria were: surgery in the mouth or throat area, nasal intubation, the use of oesophageal probe, ongoing upper airway infection and expected duration of surgery >240 minutes.

### Anaesthesia

The anaesthesia protocol followed hospital routines and could include maintenance of anaesthesia using either target- controlled infusion (TCI) with propofol and remifentanil or inhalation anesthesia with sevorane or desflurane in oxygen and air. Standard monitoring included oxygen saturation, heart rate, non- invasive blood pressure, end-tidal carbon dioxide concentration, bispectral index in patients having TCI and neuro- muscular transmission monitoring in patients given muscle-relaxant. The ETT used in this study was Mallinckrodt Hi-Contour™ (Mallinckrodt, Athlone, Ireland) while a disposable LMA (Unique™, Shanghai, China) was used in almost all cases, except in a few patients where a non- disposable Proseal (Seychelles) was used.

### Insertion of the laryngeal mask airway and endotracheal intubation

The insertion technique for the LMA was left to the discretion of the personnel using the device. A water-based lubricant (Glidslem APL, Stockholm, Sweden) was used on the posterior and lateral surfaces of the LMA cuff but not on the ETT cuff. Air was used to ensure adequate sealing of the cuff using existing hospital routines so that the cuff pressure was maintained at 20–30 cm H_2_O in an ETT [16] and at 30–60 cm H_2_O in the LMA [17]. Cuff pressure was measured continuously during surgery and documented regularly in the anaesthesia chart.

### Measurement and recordings

In addition to recording the included patient’s age, height and weight, and the type of surgery, the following additional measurements were made: anaesthesia experience of the anaesthetist (years) and insertion technique (LMA cuff inflated or partially deflated). Furthermore, ease of LMA insertion was graded into easy (one attempt, no tactile resistance), some difficulty (one attempt but tactile resistance) and difficult (two or more attempts) [18]. Finally, the number of attempted laryngoscopies (one, two or ≥ three), the use of extra airway management equipment during intubation (stylet or video laryngoscope), and fixation of the ETT (left or right cheek, or in the middle of the mouth) were also recorded. Drugs that were administered during anaesthesia and that may affect POST or PH such as non-depolarizing or depolarizing muscle relaxant, cortisone, opioids and non-steroidal anti-inflammatory drug were also recorded.

### Procedure

One of the authors (MJ) or the research nurses performed the follow-up questions. Standardized questions were presented to the patients and data collected from all patients in the post-anaesthesia care unit (PACU), and 24 hours after extubation. The 24 hour follow- up of inpatients was performed in the ward while ambulatory surgery patients were interviewed by a telephone call. If the patient reported any symptom at 24 hours, assessment was done once each day at 48, 72 and 96 hours until the patients were symptom-free. The patients were asked if POST was present at rest, which was defined as constant pain in the throat, or if POST increased on swallowing or talking?

Patients having symptoms for longer than 96 hours were offered telephone advice by an anaesthesiologist. If the patient had symptoms at 96 hours, a follow-up call was made after five to seven days by one of the investigators (MJ).

### Postoperative sore throat and hoarseness

The scale used to evaluate POST was a four graded scale: 0 = No sore throat, 1 = Mild sore throat (less than with a cold), 2 = Moderate sore throat (as with a cold) and 3 = Severe sore throat (more severe than with a cold) [7]. PH was scored as: 0 = No hoarseness, 1 = Mild hoarseness (noticed by the patient only), 2 = Severe hoarseness (noticed at the time of the interview by the personnel) and 3 = Aphonia (inability to speak) [19]. Both scales are thought to be reliable and have been used in several studies [15,20-22].

An open-ended question was also used to assess POST: “*Can you describe the symptom in your own words”.*

### Location of postoperative sore throat

**Figure 1:**
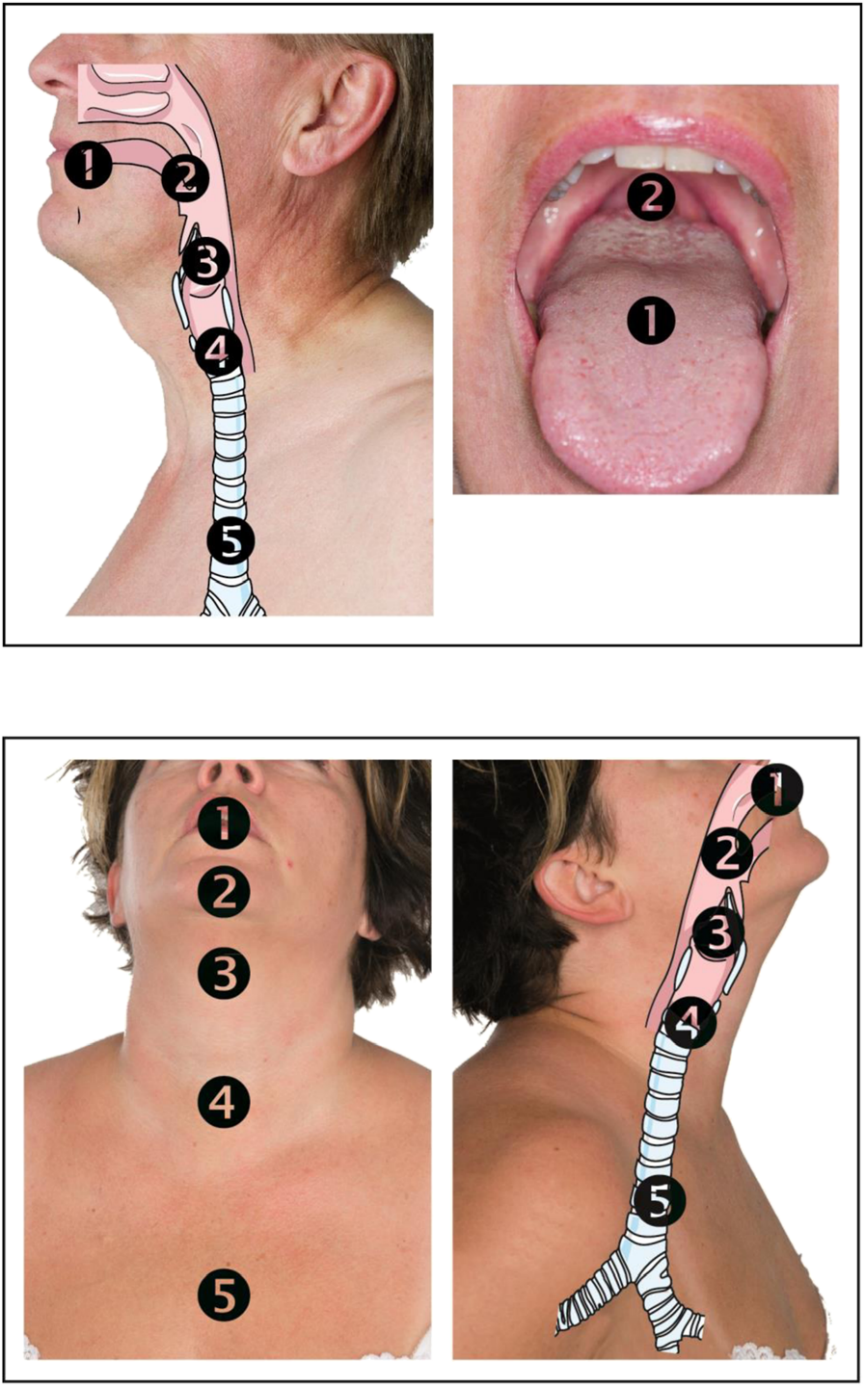
**A two-sided laminated photograph for localization of the sore throat** ©**.**

The patients assessed the location of sore throat in the PACU and at 24 hours follow up, using a photograph showing the exterior and interior of the throat and mouth in the following way: 1 = in the mouth, 2 = in the pharynx, 3 = above larynx, 4 = below larynx, 5 = high up in the chest (Figure 1). Verbal and written consent were obtained to publish the photographs of the persons included.

### Statistics

A *priori* sample size was decided after reviewing the literature. The studies available were not consistent in reporting the incidence of POST and potential differences between the sexes after an LMA [18,23-25] or an ETT [2,7,26,]. From these studies, we determined that the incidence of POST at the PACU was 40% when all airway devices were pooled together. Our hypothesis was that men have a lower incidence of POST, approximately 20% (absolute difference = 20%). Assuming a power of 80% and a significance level of 0.05 we determined that 91 patients would be needed/group. The study was also set to be ongoing for four months. Uni- and bi- variate analyses between gender and subgroup analysis between LMA and ETT were performed. In order to test for normal distribution, a one sample Kolmogorov Smirnov analysis was used. For comparison of demographic data and analgesic consumption the Student’s *t-* test or Mann–Whitney *U* test was used as appropriate. Differences between groups for dichotomous data were analyzed using the Chi-square-test or the Fisher’s exact test. All statistical analyses were performed with SPSS 17.0 for windows software (SPSS Inc. Chicago, IL, USA). P < 0.05 was considered statistically significant.

## Results

**Figure 2:**
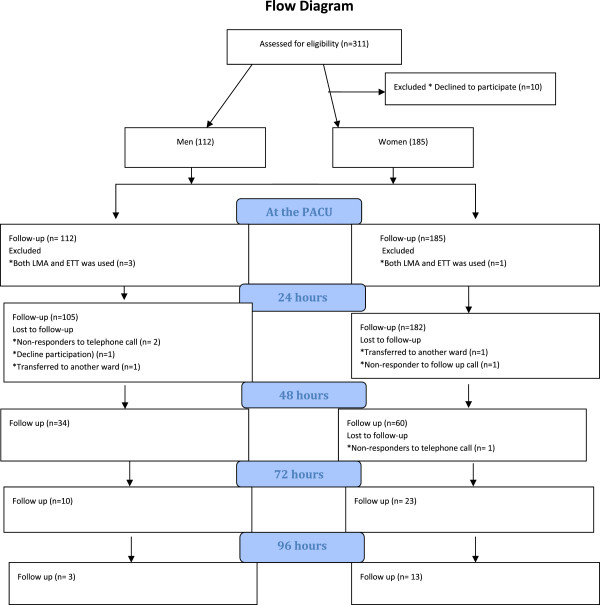
Number of patients contacted and evaluated through the study.

During the period January to April 2013 a total of 301 patients, (men n = 115 and women n = 186) from five different surgical departments (Urology, General-, Orthopedic-, Gynecological- and Hand- surgery) were consecutively screened and enrolled in the study. Four patients were subsequently excluded from the analysis since they had both an LMA and an ETT during anaesthesia. In one man, several unsuccessful attempts were made to insert a Proseal LMA and he was subsequently intubated. Two men and one woman had to be intubated during surgery because of in adequate ventilation when using an LMA. Therefore, a total of 297 patients were included in the final analysis (Figure 2). Men weighed more (p < 0.001) and were taller (p < 0.001) and required more intraoperative fentanyl (p = 0.008). There were no gender differences in morphine and ketobemidone consumption at the PACU (Table 1).

**Table 1:** Patients characteristics and demographic data

	**Men (n = 112)**	**Women (n = 185)**	**P-value**
**Patient characteristics**			
Age(yr)	56(16)	53(18)	p = 0.85^Ω^
ASA I/II/III n (%)	50(45)/50(45)/11(10)	85(46)/91(49)/8(0.5)	p = 0.3^∞^
Height (cm)	179(7)	165(6)	p = <0.001^Ω^
Weight (kg)	87(16)	71(14)	p = <0.001^Ω^
**Perioperative medications**			
Rocuronium	52(24)	42(17)	p < 0.001§
Fentanyl	157(86)	127(100)	p = 0.008§
Remifentanil	34(195)	136(359)	p < 0.001§
Ketobemidon	0.08(0)	0.7(0)	p < 0.001§
Bethametasone	0.4(1.3)	2(2.3)	p < 0.001§
Morphine at the PACU	4.8(16)	1.9(3.5)	NS
Ketobemidon at the PACU	3(16)	0.5(1.5)	NS
**Perioperative characteristics**			
Hand Surgery n (%)	19(17)	20(11)	
General Surgery n (%)	23(20)	65(35)	
Gynaecology n (%)		44(24)	
Orthopaedics n (%)	35(31)	48(26)	
Urology n (%)	35(31)	8(4)	
Daysurgery/Inpatients n (%)	55(49)/57(51)	107(58)/78(42)	p = 0.1^∞^
Duration of surgery (min)	67(10–203)*	59 (6–234)*	p = 0.02^§^
ETT size 6.0/7.0/8.0 n(%)	0/2(3)/59(97)	78(89)/10(11)/0	NA
LMA size 3/4/5 n(%)	0/1(2)/50(98)	5(5)/92(95)/0	NA
Blood on the device n(%)	8(7)	9(5)	p = 0.5^∞^
Experience of the anaesthesia staff (yr)	6(0–39)*	4(0–39)*	p = 0.1^§^
Time at the PACU (min)	180(60–2160)*	192(45–620)	p = 0.7^§^

All results are presented as mean (SD) and median (range)*. Analyzed with the Mann–Whitney U –test^§.^ Analysed with Chi-square^∞^. Analysed with Students T-test^Ω^. PACU = Postanaeshesia care unit. LMA = Laryngeal mask airway. ETT = Endotracheal tube. NA = Not applicable.

The number of attempts needed to intubate men and women respectively were as follows: one attempt in 32(29%) and 72 (39%), two attempts in 21 (19%) and 15(8%) and three or more attempts in 6 (6%) and 0 (p = 0.002). A stylet was used to aid insertion in more men compared to women 12 vs. 7 (63% vs. 32%; p = 0.017) .

The LMA was used in 134 (91%) and Pro Seal in 13 (9%) of patients. The registered nurse anaesthetist or anaesthesiologist chose the size of LMA from personal experience and using the guidelines recommended by the manufacturers. There were no gender differences in how the LMA was inserted *i.e.* with the cuff partially inflated or deflated cuff (p = 0.3). Rotation of the LMA during insertion was more common in men (p = 0.001). The LMA insertion was graded in women as follows; easy 82% (n = 77), some difficulty 15% (n = 14) and difficult 3% (n = 3). Corresponding values for men were, easy 80% (n = 41), some difficulty 12% (n = 6) and difficult 8% (n = 4). Even though insertion was considered easy, more women had POST compared to men (p = 0.07). Irrespective of airway device (ETT or LMA) used there were no significant gender difference in POST or PH at the PACU. When stratified for different surgical departments, irrespective of airway device, there were more women who had POST after hand-, orthopedic-, gynecology and general surgery. However, more men had POST after urology surgery. There were no significant differences between women and men after an ETT (27% vs. 38%, p = 0.2 ). However, there were more women than men who had POST after an LMA (26% vs. 6%, p = 0.004). If the Proseal LMA was removed from the analysis there were more women than men who had POST (24% vs. 5%, p = 0.004) and there were no significant difference in PH between genders. More women (n = 11) than men (n = 1) said that POST was present during swallowing after an LMA (Table 2). There were no significant differences in PH after either an ETT or an LMA between men and women. There was a higher incidence of POST in the ETT group compared to the LMA group (32% vs. 19%, p = 0.012) as well as a higher incidence of PH in the ETT group compared to the LMA group (57% vs. 33%, p < 0.001) in the PACU.

**Table 2:** Pain in the throat

	**In the PACU**		**After 24 hours**	
	**LMA**	**ETT**	**LMA**	**ETT**
	**(n = 24)**	**(n = 45)**	(n = 18*)	(n = 33**)
Pain during rest	0	1(2)	1(5)	0
Men/Women (n)		0/1	0/1	
Pain during speech	1(4)	0	0	0
Men/Women (n)	1/0			
Pain during swallowing	12(50)	12(27)	7(39)	14(42)
Men/Women (n)	1/11	7/5	0/7	9/5
Pain at rest, during speech, and swallowing	9(38)	19(42)	7(39)	12(36)
Men/Women (n)	1/8	10/9	3/4	6/6
Pain during speech and swallowing	2(8)	8(18)	3(17)	5(15)
Men/Women (n)	0/2	4/4	0/3	0/5
Pain at rest and during speech	0	3(6)	0	0
Men/Women (n)		1/2		
Pain at rest and during swallow	0	2(4)	0	2(6)
Men/Women (n)		1/1		0/2

Presented as n (%). *Two missing. **One missing.

LMA = Laryngeal Mask Airway. ETT = Endotracheal tube. PACU = Post Anaesthesia Care Unit.

### Location of POST

There were no significant differences between genders in location of POST after an ETT. Of those patients who reported pain in the PACU, the location of the sore throat was below the larynx after an ETT in 24% (n = 11) vs. 4% (n = 1) after an LMA. In patients with sore throat above the larynx, the distribution of symptoms between ETT vs. LMA was as follows: in the pharynx, 22% (n = 10) vs. 37% (n = 10), above the larynx, 37% (n = 17) vs. 52% (n = 14) and finally top of the chest, 2% (n = 1) vs. 4% (n = 1) respectively. A few patients reported pain at two levels. In the pharynx and above the larynx after an ETT vs. LMA, 11% (n = 5) vs. 4% (n = 1) and above and below the larynx in 4% (n = 2) patients who had an ETT.

When the patients were asked to describe the feeling in their throat in the PACU the most common words used were: dryness in the throat, feels like a cold, burning pain, irritation or tenderness and a swollen feeling.

### Follow-up

**Figure 3:**
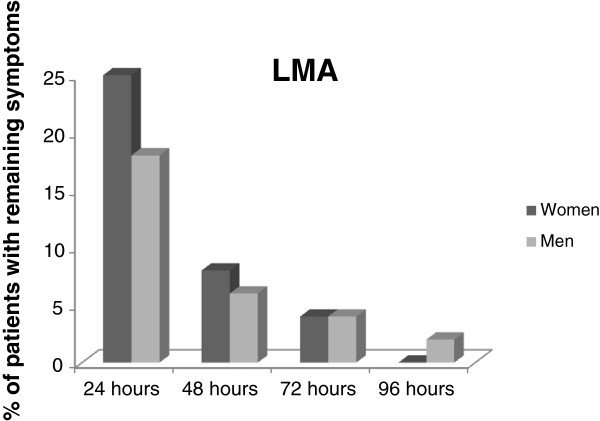
Men and women with remaining postoperative sore throat and/or hoarseness after an LMA.

**Figure 4:**
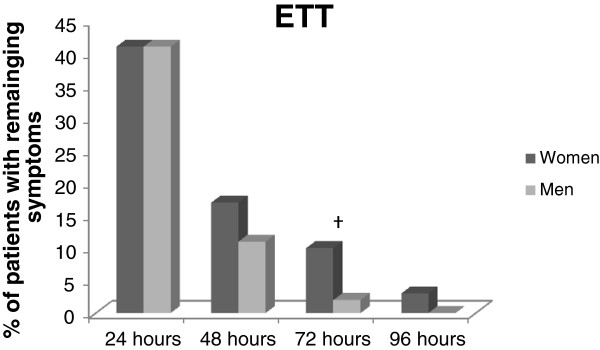
**Men and women with remaining postoperative sore throat and/or hoarseness after an ETT. **^†^Significantly more women than men had remaining symptom p=0.04^^Analyzed with Chi-square. Abbreviations: ETT= Endotracheal tube.

Irrespective of airway device used there were no significant gender differences at any time during follow up. Also, there were no significant gender differences in remaining symptom of either POST or PH after an LMA (Figure 3), and no significant gender differences after an intubation, except during 72 hours- follow up when significantly more women had POST or PH (p = 0.04) (Figure 4).

## Discussion

Our main findings were that no statistically significant differences in POST and PH were found, irrespective of gender (male or female) or type of airway used (LMA or ETT). This shows that postoperative sore throat and hoarseness are a concern for the patient regardless of whether an ETT or an LMA was used. Also, there were no significant gender differences in POST after an ETT. However, more women had POST after an LMA than men. Finally, no statistically significant differences in PH were found between genders or the type of airway used.

This study is one of few studies that did not find any gender differences in POST after an ETT [15,26]. One reason for this finding could be that there is some evidence in the literature that a smaller sized ETT in women decreases POST [12,27,28]. In our present study, most women had a small ETT, which may consequently have reduced the incidence of POST in women. However, we did find that women have more POST after an LMA than men, which confirms the findings of Nott *et al.*[25], but not Grady *et al.*[24] who found that men and women had a similar incidence of POST (20%). The low incidence of POST in men in our study compared to the other studies raises questions as to why men and women respond differently to a standard LMA. Despite the manufacturers recommendation many anaesthetists inserted the LMA with a partially inflated cuff. Having said this, earlier studies have not confirmed that the insertion technique is associated with POST [29,30]. The anatomy of the pharynx differs between the sexes [31] and it is possible that currently used LMAs are devised to better suit the anatomical features of the male pharynx rather than the female and therefore the lower incidence of POST in men. Another possible confounder in this study could be the wide range of cuff pressure in the LMAs. The reason for following the manufactures guidelines were because of the lack of consensus amongst researchers in an association between cuff pressure and POST. In the study by Burgard *et al.*[32], the cuff pressure in the control group was not adjusted during surgery and it became as high as 200 cm H_2_O while the cuff pressure was held below 60 cm H_2_O intra-operatively in the intervention group. However, in the study by Rieger *et al.*[33], there were no significant differences between intervention and the control group when the cuff pressure was at 30 mmHg (41 cm H_2_O) and 180 mmHg (244 cm H_2_O) respectively. A recently published study showed that a cuff pressure at 60 cm H_2_O compared to 25 cm H_2_O in LMA Supreme can increase pharyngolaryngeal adverse events. However, this study made no comparison between genders [34]. Although we used a smaller LMA in women than men, the anatomical formation of the LMA rather than its size may play an important role in the development of POST. We also found a higher incidence of PH in our patients after an LMA compared to the study by Yu *et al.*[1]. In one previous study, the authors suggest that *a* larger LMA (size 5) may contribute to an increase in the frequency of PH in men. However, the LMA size (3 or 4) was not associated with a higher incidence of PH in women [24]. Whatever the reason, patients are less likely to be satisfied with anaesthesia if they have PH [10,23]. The etiology of PH may differ between airway devices, *e.g.* after an ETT it may result from direct trauma to the vocal cords causing edema [35], while the cuff in the LMA may cause compression of the laryngeal nerve [36]. It is, however, important to point out that all patients in our department receive oxygen via nasal prongs in the PACU, which may also have contributed to the symptoms of both PH and POST because of the high flow of dry oxygen.

Persistent symptoms can be a constraint for patients, and one cause of patient dissatisfaction. In our previous study, 10% of the patients had persistent symptoms of POST and/or PH 96 h after an ETT [28], which contrasts with only 3% of the women who had either POST/PH after 96 hours in the present study. Thus, persistent symptoms of POST/PH after 96 hours, seem to occur in only a small number of patients and all patients were symptom-free approximately two weeks after the anaesthetic in this study.

Research investigating gender difference in anaesthesia revealed that women have a different recovery profile than men [37]. Despite the fact that women emerged faster from general anesthesia [37,38], their Quality of Recovery was poorer than that of men. In this study, however, there seem to be no significant differences between men and women in the recovery from POST and PH after LMA or ETT. However, there were significant differences between the sexes in pain medication peroperatively. Men received higher dosage of opioids than women which could be due to the calculation of doses in μg/kg. Cortisone intravenously is known to decrease POST in the postoperative period [39,40]. In this study, betamethasone was used for decreasing postoperative nausea and vomiting (PONV). Bethamethasone are commonly used in women who are prone to suffer from PONV [41]. The use of betamethasone for preventing postoperative nausea may influence the development of POST.

To the best of our knowledge only Joorgensen *et al.*[42] have studied the location of POST as assed by the patient. One reason for not asking about the precise location of POST may be that it is difficult to explain correctly. Despite the complexity of pain localization, we did find that our patients seem to be able to localize pain in the throat. We found that more patients located the pain below the larynx after an ETT compared to an LMA, probably due to the design and shape of the cuff in the ETT [43]. Pain above the larynx, was more common after an LMA than an ETT (52% vs. 37%), which is likely because the LMA cuff exerts pressure on the mucosa above the larynx [18]. However, pain above the larynx after an ETT could also be caused by laryngoscopy [15].

Pain in the throat was more common when using an LMA than an ETT in women during swallowing. As far as we know, no difference between the sexes has been reported before even though the findings by Rieger *et al.* confirmed that pain during swallowing was more common after an LMA than an ETT [23]. There are also many patients who experienced pain in the throat in the PACU at all time, namely, during rest, swallowing and talking in this study.

The follow-up questions were asked in a non-leading but direct manor by trained personnel. Direct questioning is known to increase the number of events reported by patients, which may explain the high incidence of PH in our study [44]. According to Myles *et al.* there may be a gender difference in the ability to report postoperative symptoms, and that women may find it easier to report symptoms to a nurse than do men [45]. If this indeed was the case in the present study remains unclear.

### Study limitations

One weakness of the present study is that the techniques for LMA insertion vary between individuals as this was not standardized. However, the strength of this study design is that the results reflect a real-life situation with no attempts to influence insertion of the LMA or to control the experience of the personnel in any way. We did not record coughing and bucking which can have affected the result. Finally, we did not register the analgesic consumption in the ward (inpatients) or home (out patients) and this may naturally have affected the outcomes of interest. However, although all patients had analgesic medication, these did not seem to relieve POST in all patients.

## Conclusions

Our study contradicts previous studies regarding the gender difference of POST and PH following general anaesthesia. In a clinical setting where women are intubated with a smaller size ETT there were no significant difference in POST or PH between genders. Additionally, more women than men have POST when an LMA is used. This is important to know so that one can take appropriate steps to prevent the development of sore throat or hoarseness in men and women.

## Competing interests

The authors declare that they have no competing interest.

## Authors’ contribution

Substantial contribution to conception and design (MJ, AG, UN). Acquisition of data, or analysis and interpretation of data (MJ,UN). Drafting the article or revising it critically for important intellectual content (MJ,AG, UN). Final approval of the version to be published (MJ,AG,UN).

## Pre-publication history

The pre-publication history for this paper can be accessed here:

http://www.biomedcentral.com/1471-2253/14/56/prepub
